# Sterile Inflammation of Brain, due to Activation of Innate Immunity, as a Culprit in Psychiatric Disorders

**DOI:** 10.3389/fpsyt.2018.00060

**Published:** 2018-02-28

**Authors:** Mariusz Z. Ratajczak, Daniel Pedziwiatr, Monika Cymer, Magda Kucia, Jolanta Kucharska-Mazur, Jerzy Samochowiec

**Affiliations:** ^1^Stem Cell Institute at James Graham Brown Cancer Center, University of Louisville, Louisville, KY, United States; ^2^Department of Regenerative Medicine, Warsaw Medical University, Warsaw, Poland; ^3^Department of Psychiatry, Pomeranian Medical University, Szczecin, Poland

**Keywords:** sterile inflammation, complement cascade, stem cell mobilization, mannan-binding lectin, heme oxygenase 1

## Abstract

Evidence has accumulated that the occurrence of psychiatric disorders is related to chronic inflammation. In support of this linkage, changes in the levels of circulating pro-inflammatory cytokines and chemokines in the peripheral blood (PB) of psychiatric patients as well as correlations between chronic inflammatory processes and psychiatric disorders have been described. Furthermore, an inflammatory process known as “sterile inflammation” when initiated directly in brain tissue may trigger the onset of psychoses. In this review, we will present the hypothesis that prolonged or chronic activation of the complement cascade (ComC) directly triggers inflammation in the brain and affects the proper function of this organ. Based on the current literature and our own work on mechanisms activating the ComC we hypothesize that inflammation in the brain is initiated by the mannan-binding lectin pathway of ComC activation. This activation is triggered by an increase in brain tissue of danger-associated molecular pattern (DAMP) mediators, including extracellular ATP and high-mobility group box 1 (HMGB1) protein, which are recognized by circulating pattern-recognition receptors, including mannan-binding lectin (MBL), that activate the ComC. On the other hand, this process is controlled by the anti-inflammatory action of heme oxygenase 1 (HO-1). In this review, we will try to connect changes in the release of DAMPs in the brain with inflammatory processes triggered by the innate immunity involving activation of the ComC as well as the inflammation-limiting effects of the anti-inflammatory HO-1 pathway. We will also discuss parallel observations that during ComC activation subsets of stem cells are mobilized into PB from bone marrow that are potentially involved in repair mechanisms.

## Introduction

In this review, we will discuss the emerging picture of the interplay between the stress- or inflammation-mediated activation of innate immunity, inflammation, and changes in the levels of inflammation markers and stem cells circulating in peripheral blood (PB), providing a mechanistic basis for the occurrence of psychiatric disorders.

It is well known that patients suffering from psychotic disorders often exhibit inflammation-related abnormalities in PB, including (i) elevated levels of circulating pro-inflammatory cytokines and chemokines, (ii) increased numbers of circulating monocytes and neutrophils, as well as (iii) enhanced reactivity of microglia, astrocytes, and endothelial cells to various pro-inflammatory signals (1–4). Furthermore, severe depression is often comorbid with chronic inflammatory conditions, and patients with pre-existing inflammatory diseases are more susceptible to developing mood disorders than healthy individuals (5, 6). These findings support the existence of crosstalk between the central nervous system (CNS) and innate and adaptive immunity, which can be explained, at least partially, by the co-evolution of both systems.

The main role of innate immunity, which arose during evolution ~600 million years ago, is to detect specific molecular patterns present in invading microorganisms or in the host organism’s own damaged tissues but not in healthy tissues (1–4). Therefore, the innate immune system mediates inflammation as a physiological response to (i) insult, (ii) infection, and (iii) biological stress. By contrast, adaptive immunity appeared later, ~500 million years ago, and unlike innate immunity depends on adaptive responses against antigens that have been recognized and subsequently presented to adaptive immunity cells by the innate immunity (1, 4). The CNS arose ~550 million years ago, and thus both nervous and innate and adaptive immune systems have co-evolved during evolution and are in constant crosstalk and communication (1, 3, 4). Therefore, one can envision that understanding and controlling these mutual interactions between the immune and nervous systems could be a fundamental step in preventing some CNS diseases, including cerebral neuropathies (bipolar disorder, schizophrenia, major depressive disorder, autism, Alzheimer’s disease, Parkinsonism, epilepsy, and migraine). This possibility will be discussed in this review in the context of innate immunity-mediated inflammatory processes and the anti-inflammatory negative-feedback loops maintained by heme oxygenase 1 (HO-1). This enzyme is activated in response to inflammation to inhibit the pro-inflammatory action of the complement cascade (ComC) (7, 8).

We will explore connections between changes in the release of danger-associated molecular pattern molecules (DAMPs) with aberrant ATP-mediated purinergic signaling occurring in the brain, sterile inflammation triggered by the innate immunity involving activation of the ComC and coagulation cascade (CoaC), and the inflammation-limiting effects exerted by HO-1. In parallel, we will discuss results indicating that stem cells are mobilized from bone marrow (BM) into PB as a result of ComC activation (9–12), which might be potentially involved in certain repair mechanisms in the CNS.

## Activation of Innate Immunity in Response to Stress and Inflammation

Innate immunity, which is also sometimes called non-specific or in-born immunity, developed early in evolution and plays an important role as a mechanism to regulate (i) the response to invading pathogens, (ii) tissue and organ injury, (iii) the response to biological stress, and (iv) tissue and organ development and regeneration (12). Nevertheless, the innate system does not provide long-lasting immunity to the host. Rather, this role is assigned to the adaptive immune system (1, 2). However, the most important role of innate immunity is still as a pivotal subsystem of the overall immune response.

Innate immunity consists of (i) the ComC proteins present in biological fluids usually in inactive form to become activated in a cascade type of amplifying reactions triggered by classical-, alternative- or mannan-binding lectin pathway and (ii) several types of cells, such as phagocytic cells (macrophages and neutrophils), mast cells, eosinophils, basophils, dendritic cells, natural killer cells, and γ/δ T cells. These latter cells exhibit several characteristics that place them on the border between innate and adaptive immunity (13). All these cell types function within the immune system to identify and eliminate invading pathogens. An important component of innate immunity are also naturally occurring antibodies (NAbs), which are produced without any previous foreign antigen exposure, infection, vaccination, or passive immunization (1, 14). These antibodies are not primarily restricted to protecting the host from invading pathogens, and their physiological role is restricted to being key regulators in recognizing neo-epitopes exposed on the surface of damaged cells. In particular, NAbs are involved in eliminating damaged cells from the tissues (14). Such a situation occurs, for example, in sterile inflammation when reactive oxygen species (ROS) are released from neutrophils or macrophages to expose neo-epitopes on the surface of target cells, and these sites are recognized and bound by NAbs from the IgM class (15).

The innate immune system maintains homeostasis in an adult organism by activating the ComC to (i) promote clearance of antibody complexes or dead cells, (ii) identify and remove pathogens and foreign substances present in tissues and biological fluids, (iii) recruit immune cells to sites of infection and tissue damage through the production of specialized mediators, such as chemokines, cytokines, and bioactive lipids, and the release of extracellular nucleotides, and (iv) activate the adaptive immune system through a process known as antigen presentation to B and T lymphocytes to elicit long-lasting memory against antigens (1). Innate immunity overlaps also with coagulation and the fibrinolytic system, as several products originating during coagulation and fibrinolysis activate the ComC (1, 16). As an example, thrombin generated from prothrombin has C5 convertase activity and cleaves C5 into the potent anaphylatoxins C5a and _desArg_C5a (16).

Also important for the main topic of this review is the response of the innate immunity network to “sterile inflammation,” which occurs, for example, during prolonged biological stress even without exposure to foreign antigens. Evidence has accumulated that this process may be initiated by pattern-recognition receptors (PRRs) and can also be activated by non-microbial signals such as DAMPs, including ATP (1–4, 17). This type of response is modulated by neural circuits that control production of immune mediators. The potential involvement of sterile inflammation in psychiatric disorders will be discussed below in more detail.

## Mechanisms of Sterile Activation of Innate Immunity

As mentioned above, more light has been shed on the nature of the host innate immune response to invading microorganisms that involves PRRs. It has been understood that the same PRRs that recognize foreign antigens can also recognize non-microbial DAMP signals, such as those secreted by cells, including extracellular ATP and high-mobility group box 1 (HMGB1) protein (2, 18–20). If occurring in the CNS, this mechanism may trigger sterile inflammation, which may potentially lead to psychiatric disorders (1–4). The leading role in this mechanism is played by the secretion of DAMPs from activated microglia and astrocytes in brain tissue or from circulating innate immune cells (e.g., granulocytes and monocytes). In fact, it has been reported that the number of granulocytes and monocytes circulating in PB increases in patients suffering from psychotic disorders (2–4), and, in parallel, microscopic analysis has revealed the presence of activated microglia and astrocytes in the brains of psychotic patients (3).

A potential mechanism leading to an increase in DAMP secretion, which initiates sterile brain inflammation by activating the ComC in a mannan-binding lectin (MBL)-dependent manner, involves the aberrant function of pannexins (21). These proteins are distant homologs of connexins; while unable to form gap junctions they form transmembrane channels on the cell surface, releasing molecules such as ATP from activated cells (20). ATP is well known as a ubiquitous intracellular molecular energy source but may also be secreted through pannexin channels into the intercellular space, where it acts as an important signaling molecule (2, 20, 21). In addition, ATP can be also secreted into intracellular space by extracellular microvesicles (17).

In physiological situations, ATP is involved in evoking purinergic signaling in neural tissue by interacting with the G protein-coupled P2Y and the ligand-gated ion channel P2X purinergic receptors. As part of purinergic signaling in the CNS, ATP released from the synaptic terminals *via* pannexin channels binds to members of both the P2X and P2Y receptor families (22). This interaction plays an important role in activating neurons and at the same time is involved in neuronal–glial communication, as microglia highly express both types of receptors. It is also well known that both ATP and its degradation product adenosine induce microglia and astrocyte proliferation and activation (3). Therefore, the pannexin channels, in particular pannexin-1, is an integral component of the P2X/P2Y purinergic signaling pathway, which, along with released ATP and ectonucleotidases that mediate the processing of ATP to ADP, AMP, and adenosine in the extracellular space, may contribute to several feedback loops during the inflammatory response in the CNS (22).

**Figure 1:**
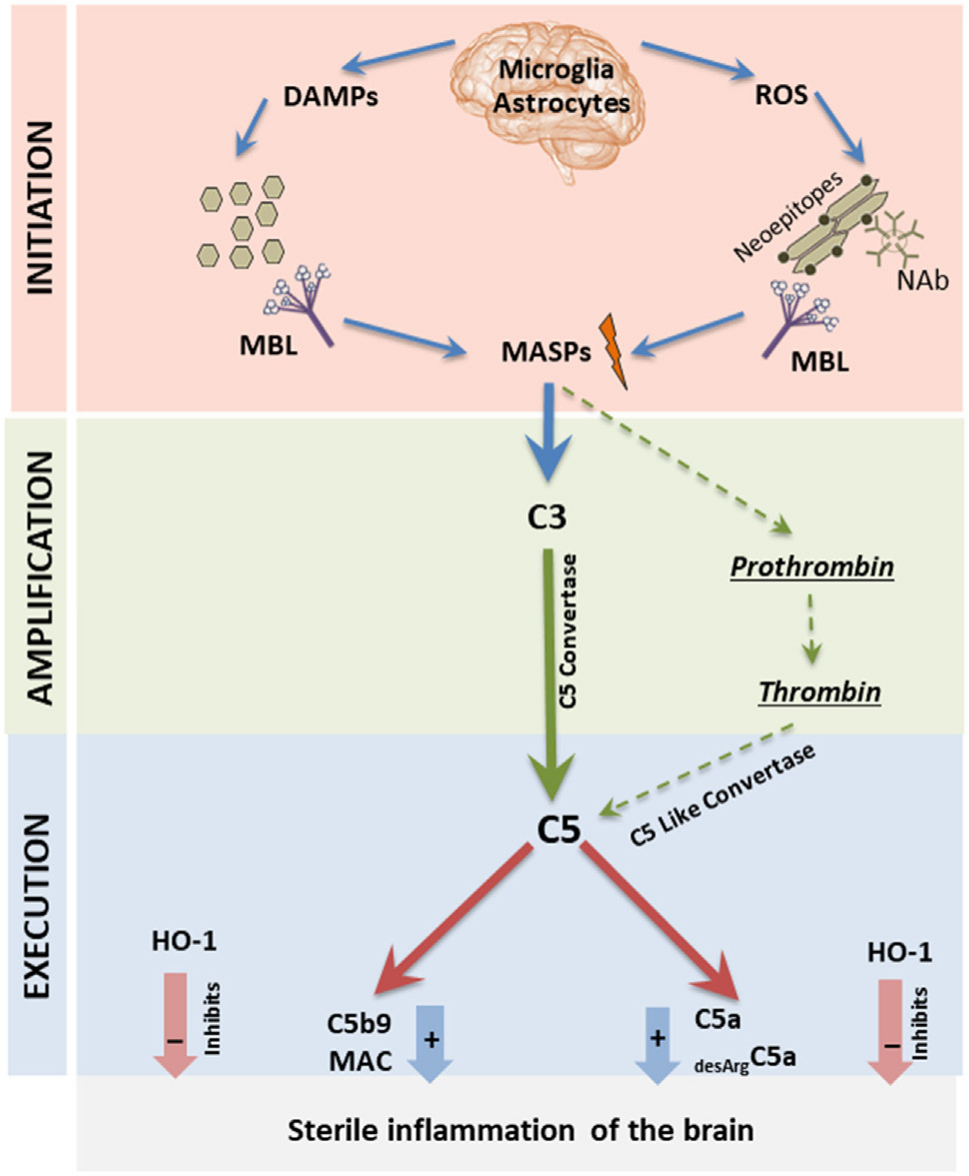
Proposed by us MBL-induced three-step model for triggering sterile inflammation in the brain tissue. All three phases of the complement cascade (ComC) activation process (initiation-, amplification-, and execution phase) are depicted here. In Step I (initiation phase), activation of microglia, astrocytes, and monocytes induces the release of danger-associated molecular pattern molecules (DAMPs) (ATP and high-mobility group box 1), and the secretion of ROS by these cells exposes neo-epitopes. Both DAMPs and neo-epitope–IgM complexes are recognized by mannose-binding lectin (MBL), which activates the ComC and CoaC in a MASP-1 and -2 dependent manner in Step II (amplification phase). In Step III (execution phase), the C5 cleavage fragments anaphylatoxins C5a and desArgC5a promote sterile inflammation in the brain, and this process is negatively regulated by heme oxygenase 1 (HO-1).

What is relevant for this review, pannexin 1 is highly expressed in brain tissue and releases ATP, an important DAMP molecule recognized by circulating MBL, into the extracellular space and thus is able to activate the mannan-binding lectin pathway of ComC activation (Figure 1). Another important DAMP molecule is the HMGB1 protein, which is also recognized by MBL and may trigger activation of the ComC as well (17, 20). In fact, in patients suffering from psychiatric disorders, an increase in the release of both ATP and HMGB1 into the extracellular space of brain tissue has been reported (3, 21). This increase in the DAMP level in brain may be primarily a result of sterile brain inflammation or a secondary effect due to certain systemic inflammation disorders (e.g., lupus erythromatosus and arthritis) (4).

As mentioned, both ATP and HMGB1 molecules are recognized by a major PRR of the innate immune system, MBL (Figure 1). Once bound to these ligands, MBL recruits MBL-associated serine proteases (MASP-1 and -2) and mainly MASP-1 initiates activation of the ComC by involving the mannan-binding lectin pathway (19, 20). As mentioned above, activated microglia cells may also release ROS that, by oxidation of cell membranes, exposes neo-epitopes recognized by naturally occurring antibodies (Nabs) of the IgM class (14, 15, 20), and such neoepitope–Nabs complexes trigger activation of the ComC *via* MBL–MASP interactions (Figure 1). Beside a major role of ATP and HMGB1 in initiating sterile inflammation, there are also involved other DAMPs, such as DNA, RNA, hyaluronan fragments, uric acid, heparin sulfate and S100 proteins (2).

MBL-recruited MASPs cleave C3 and C3 cleavage products and initiate the emergence of classical C5 convertase, which subsequently cleaves C5 into the anaphylatoxins C5a and iC5b (1, 18). In parallel, MASP-1 also activates prothrombin, giving rise to thrombin (16), which has, as mentioned above, C5 convertase-like activity (Figure 1). Activation of ComC during sterile inflammation may be also additionally augmented by action of alternative pathway of ComC activation (1, 2). Overall, ComC can be activated during sterile inflammation in acute as well chronic way. The level and duration of activation may affect intensity of observed symptoms. The end products of ComC activation, the anaphylatoxins C5a and desArgC5a as well as C5b9 [also known as the membrane attack complex (MAC)] perpetuate inflammation in the surrounding tissues by activating cells through interaction with specific surface receptors present e.g., on microglia cells and astrocytes (C5a, desArgC5a) or even damage cells in the brain (MAC). This process occurring in brain as part of prolonged sterile inflammation may lead to psychotic disorders. In support of this notion, we have already reported that the ComC becomes activated in patients suffering from psychotic disorders (9–12). Furthermore, in cases in which the brain is exposed to activated ComC mediators circulating in blood due to a systemic disorder, these mediators may penetrate the blood–brain barrier, particularly when it is damaged and permeabilized, and thereby affect neural tissue (2–5).

Overall, in this proposed scenario, ComC cleavage fragments, such as the C5a and _desArg_C5a anaphylatoxins, activate microglia, astrocytes, and endothelial cells in brain tissue. The most important targets are microglia cells, also known as resident brain macrophages, which account for up to 15% of all cells found within the brain and as widely accepted act as the first and main form of active immune defense in the CNS (23). They may be responsible for the release of DAMPs as well as several pro-inflammatory cytokines, chemokines, and bioactive lipids. In parallel, systemic activation of the ComC may also lead to release of all these factors from neutrophils and macrophages residing in BM, spleen, and other organs. However, this process needs to be controlled, and there are several limiting mechanisms that rein in an activated inflammatory reaction. This issue will be addressed below with respect to the potential modulation by HO-1 activity (7, 19, 24).

## Mechanisms That Limit Sterile Inflammation in Brain Tissue

Given the possible role of inflammation in the pathogenesis of psychotic disorders, clinical trials have been initiated to inhibit inflammation in psychotic patients, and an example of such a treatment is the application of omega fatty acids and the cyclooxygenase-2 inhibitor celecoxib in schizophrenia patients (25). The potential armamentarium of anti-inflammatory drugs that could be used for treatment is the subject of a recent comprehensive review (3).

However, given the leading role of ComC activation in the CNS during sterile inflammation, one might also consider more ComC-directed treatment strategies. One of the most important enzymes that counteracts ComC-mediated inflammation is, as mentioned above, HO-1 (7, 19, 24). To support this, we have demonstrated that upregulation of HO-1 activity by small molecular activators inhibits activation of ComC in BM (19). In fact, it has been reported that low levels of activity of HO-1 is associated with depressive symptoms and may contribute to depressive and hypertensive comorbidity (24). Similarly, inducible nitric oxide synthase (iNOS) may be in certain conditions another ComC-limiting system, and its enhanced activity has been shown to exert antidepressant effects in a murine model of depression (26). Based on this finding, one can ask whether strategies for upregulating HO-1 and perhaps also iNOS could lead to beneficial effects in the treatment of psychotic patients. Similarly, there is the question of whether ComC inhibitors might find applications in cases of severe psychotic syndromes as well (1). Taking into consideration the role of defective purinergic signaling in the brain and the release of excessive ATP *via* pannexin channels, one might also consider the application of probenecid, a pannexin-1 channel blocker (27).

Further studies are also needed to determine whether defects in complement activation affect the epidemiology of psychotic disorders. Since, as proposed, the MBL ComC-activation pathway may play an important role in sterile inflammation in the brain, one can ask whether MBL deficiency has a protective effect. Interestingly, human MBL protein (MBL2) deficiency, is the most common form of ComC deficiency seen in 5–10% of the population. As reported, structural mutations in exon 1 of the human *MBL2* gene at codon 52 (Arg → Cys, allele D), codon 54 (Gly → Asp, allele B), and codon 57 (Gly → Glu, allele C) independently by disrupting protein structure reduce the overall level of functional MBL in serum (28, 29). Furthermore, MBL2 serum levels ia affected by several nucleotide substitutions in the promoter region of the human *MBL2* gene at positions −221 (X/Y polymorphism); −550 (H/L polymorphism); −427, −349, −336, del (−324 to −329), −70, and +4 (P/Q polymorphisms) (28, 29). Based on this, it remains to be determined in the future whether MBL2 would be a good biomarker to predict resistance to sterile inflammation of brain tissue. This of course requires further study.

## Circulating Stem Cells as a Response to ComC Activation

An interesting recent observation is the changes in the numbers of cells circulating in PB in psychotic patients. Besides reports on an increase in the numbers of circulating leukocytes and monocytes during exacerbation of psychotic symptoms (2–4), evidence has accumulated that there are also changes in the number of circulating stem cells (9–12). There are several types of stem cells, such as hematopoietic stem cells (HSCs), multipotent stromal cells (MSCs), endothelial progenitor cells (EPCs), and some rare very small embryonic-like stem cells (VSELs), that circulate in PB (9–12). The most numerous are HSCs. Moreover, the number of HSCs and other stem cells circulating in PB increases during inflammation, tissue and organ damage, strenuous exercise, and biological stress (30–33). The studies of changes in the numbers of circulating stem cells have already had an impact on several areas of clinical medicine, including neurology and cardiology. Based on these findings, our team became interested in the profile of circulating stem cells in patients with psychotic disorders (9–12).

**Figure 2:**
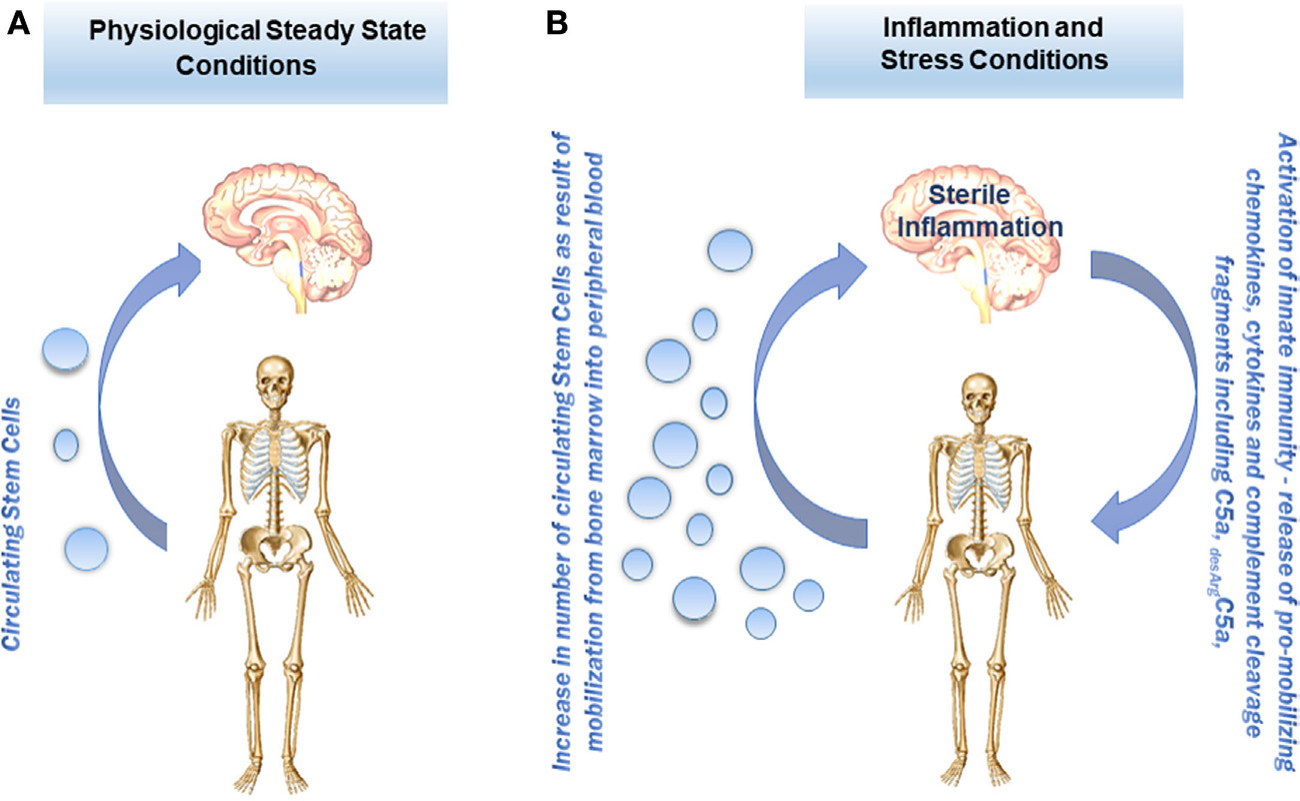
Proposed concept of stem cell trafficking between bone marrow (BM) and brain under steady-state conditions and in psychotic disorders. **(A)** Under steady-state conditions, stem cells, including hematopoietic stem cells (HSCs), multipotent stromal cells (MSCs), endothelial progenitor cells (EPCs), and the rare population of VSELs, circulate in PB at very low levels. **(B)** The numbers of these cells in PB under stress and in pathological situations increase in response to chemoattractants (e.g., sphingosine-1-phosphate, S1P; or stromal-derived factor 1, SDF-1) as well as stem cell trafficking modulators (e.g., the complement cascade (ComC) cleavage fragments C3a and C5a) that are released during the inflammation process from BM as well as from damaged brain.

We found changes in both the number of stem cells circulating in PB as well as in important factors that direct their trafficking, including ComC cleavage fragments and bioactive phospo-sphingolipids, which may be employed as diagnostic tools in psychiatry (9–12). As depicted in Figure 2, under steady-state conditions, there are always some stem cells detectable at very low levels circulating in PB, including mainly HSCs but also MSCs, EPCs, and very rare VSELs (30–33). The numbers of these stem cells increases in circulation during stress and pathological situations that are related to inflammation and tissue damage. Our team also reported that the blood plasma level of stem cell chemoattractants responsible for their egress from BM into PB (e.g., S1P and SDF-1) and ComC cleavage fragments that modulate their trafficking (e.g., C3a and C5a) increase in psychotic disorders, leading to release of these cells into the circulation (Figure 2). Interestingly, the egress of stem cells from BM into PB in most of the situations related to sterile inflammation is initiated by the MBL pathway of ComC activation (20).

We found that the pattern of cells released into the circulation as well as the profile of chemoattractants detected in PB differs between various disorders and may be potentially helpful as a diagnostic or a prognostic tool (9–12). However, we are aware that further studies are needed to understand the implications of the release of these cells from BM. There are some indications that certain processes occur during psychotic disorders that are related to brain tissue remodeling as seen for example in depression, and stem cells could play a role here (34). Circulating stem cells may also be a source of paracrine soluble factors (cytokines, growth factors, chemokines, and bioactive lipids) as well as extracellular microvesicles that may deliver their content (mRNA, miRNA, proteins, mitochondria) to the brain cells (35). There is also another question: How tight is the blood-brain barrier to circulating stem cells during sterile inflammation in the brain? On the other hand, it is known that it is possible for more differentiated cells, such as monocytes or T lymphocytes, to cross the blood–brain barrier during inflammatory processes in the CNS (4).

In future studies, it would also be interesting to see how pharmacological as well as other types of treatment in psychotic patients, such as electro- or insulin-shock therapies, affect the egress of these cells from BM into PB and potentially enforce their trafficking between BM and brain tissue. It will be also important to study in depth effect of gut microbiota on brain inflammation (36). Furthermore, it is important to mention that ATP released in the brain as DAMP and its metabolite adenosine may also directly activate P2 and P1 purinergic receptors, respectively, that if aberrantly expressed or stimulated may impact several psychiatric conditions, including major depressive disorders, schizophrenia, bipolar disorders, autism, anxiety disorders, and attention deficit/hyperactivity disorders. This purinergic receptor mediated effects activated by released in brain ATP and its metabolites become recently a topic of an excellent review (37).

## Conclusion

We propose that sterile inflammation in the brain leading to psychotic disorders may be initiated by the MBL–MASP pathway of ComC activation. This activation may be triggered by an increase in DAMP mediators, including extracellular ATP and HMGB1, which are recognized by MBL and activate the ComC (4, 20). This process is also tightly controlled by the anti-inflammatory action of HO-1 (7, 20, 38). In parallel, during ComC activation subsets of stem cells from BM are mobilized into PB that may be involved in certain brain-remodeling processes (9–12). Therefore, these observations suggest that modulating innate immune responses related to sterile inflammation will enable the development of innovative approaches to the treatment of psychiatric disorders.

## Author Contributions

This manuscript was written by MR in consultation with the rest of the authors. All authors approved manuscript.

## Conflict of Interest Statement

The authors declare that the research was conducted in the absence of any commercial or financial relationships that could be construed as a potential conflict of interest.
